# Costs and outcome for serious hand and arm injuries during the first year after trauma – a prospective study

**DOI:** 10.1186/1471-2458-13-501

**Published:** 2013-05-24

**Authors:** Hans-Eric Rosberg, Katarina Steen Carlsson, Ragnhild I Cederlund, Eva Ramel, Lars B Dahlin

**Affiliations:** 1 Department of Hand Surgery, Skåne University Hospital, Malmö, S-205 02, Sweden; 2 Department of Clinical Sciences Malmö – Hand Surgery, Lund University, Malmö, S-205 02, Sweden; 3 The Swedish Institute for Health Economics, Lund, Sweden; 4 Health Economics, Department of Clinical Sciences Malmö, Lund University, Malmö, Sweden; 5 Division of Occupational Therapy and Gerontology, Lund University, Malmö and Lund, Lund, Sweden; 6 Department of Health Sciences, Lund University, Malmö and Lund, Lund, Sweden

**Keywords:** Hand injury, HISS, DASH, EQ-5D, Costs, Health care costs, Complex injury, Nerve injury

## Abstract

**Background:**

To study costs and outcome for serious hand and arm injuries during the first year after the trauma.

**Methods:**

In patients with a Hand Injury Severity Score (HISS) > 50, DASH and EQ-5D scores as well as factors related to costs within the health care sector, costs due to lost production and total costs were evaluated. Cox-regression analysis stratifying for mechanism of injury was used to analyse return to work.

**Results:**

The majority of the 45 included patients (median 42 years 16–64) were men with severe (n = 9) or major (n = 36) injuries with different type of injuries (amputations n = 13; complex injuries n = 18; major nerve injuries/full house n = 13; burn injury n = 1). DASH and EQ-5D decreased and increased, respectively, significantly over time during one year. Total costs (+34%) and costs of lost production were highest for persons injured at work. Factors associated with higher health care costs were age >50 years (+52%), injury at work (+40%) and partial labour market activity (+66%). Costs of lost production had a significant role in total costs of injury. Patients with major injuries had longer duration of sick leave. Patients with severe injuries were more likely to return to work [(RR 3.76 (95% CI 1.38-10.22) from Cox regression, controlling for age, gender and presence of nerve injury].

**Conclusions:**

Despite the fact that work environments have constantly improved over the last decades, we found that hand injuries at work were most costly both in terms of health care and costs of lost production, although the severity, i.e. HISS, did not differ from injuries occurring at home or during leisure.

## Background

Trauma to the upper extremity is common and such patients account for 10-30% of the attendance to an Accident and Emergency department [1,2]. Most patients are young men, and the majority of hand injuries are minor or moderate [1-3]. However, hand injuries do have an impact on the patients` work capacity, daily activities and leisure activities [4]. Thereby, the injuries have a profound power on the individual subjects as well as on the society. Irrespective of the variety of severity of hand and arm injuries, substantial costs, consisting of health care costs and costs of lost production, are induced to the society [5,6]. Even if the costs of hand injuries have been the focus in previous research, the more severe ones, some of them caused by machines [6,7], may induce substantial costs due to an increased time off work and to the subsequent permanent impaired hand and arm function [8]. Our aim was to evaluate total costs and factors associated with such costs and to measure physical function (i.e. Disability of Arm, Shoulder and Hand; DASH) and quality of life (i.e. EQ-5D) in consecutively and randomly included patients for serious hand injuries.

### Patients and methods

#### Patient material

Between February 2005 and April 2007, 153 patients with a major or severe injury according to the Hand Injury Severity Score (HISS) [9] or the modified HISS [10] were treated at our department. Totally 132 fulfilled the inclusion criteria, i.e. being able to communicate in Swedish and aged between 16–65, with a major hand injury, based on Hand Injury Severity Score (HISS) with scores >100 and with a severe hand injury with scores 50–100. We randomly, based on the individual physician on call´s decision and attention, asked 54 (45%) patients to participate in a prospective study. The patients were included independently of which weekday or month they were injured (results not shown). Nine patients declined participation in the study for various reasons. The remaining 45 (34%) patients were followed for 12 months after the injury (Table 1).

**Table 1:** Patient characteristics of included and not included patients with a severe or major hand injury

	**Included**	**Not included**
	**n = 45**	**N = 87**
***Gender***		
Female/Male	9 (20)/36 (80)	12 (14)/75 (86)
***Age***		
(years)	42 (16–64)	37 (16–65)
***HISS***	154 (52–414)	82 (51–268)
***Injury severity group***		
Major	36 (80)	29 (34)
Severe	9 (20)	57 (66)
***Emergency surgery***		
(minutes)	336 (32–1879)	
***Type of injury***		
Amputation	13 (29)	
Complex	18 (40)	
Nerve/Full-house	13 (29)	
Burn	1 (2)	
***Reconstruction of blood vessels by microsurgery***		
yes	21 (47)	
no	24 (53)	
***Cause of injury***		
Crush	17 (38)	
Cut	27 (60)	
Burn	1 (2)	
***Type of work***		
Manual	28 (62)	
Non-manual	11 (24)	
Self employed	2 (5)	
Students/early retirement/long-term sick leave	4 (9)	

Results presented as n (%) or median (min-max) if not otherwise stated.

*HISS*, Hand injury severity score (see text for references).

## Methods

Demographic data were collected from the medical notes of all 132 patients. The severity of the injury was classified with HISS or with the modified HISS when applicable. At three, six and 12 months the 45 included patients answered the region-specific Disability of the Arm, Shoulder and Hand (DASH) outcome measure [11,12] and the quality of life questionnaire EQ-5D, consisting of five questions to measure health outcome. Preference-based utility values were applied [13].

The overall influence on quality of life was analysed using EQ-5D utility values at three, six and 12 months. In a secondary analysis, a hand surgeon (HER) mapped DASH scores to EQ-5D dimensions at 12 months. DASH-derived EQ-5D utility values were compared to direct EQ-5D based values in an exploratory analysis.

Patients were classified as “returned to work” when they had resumed at least some labour market activity.

### Costs

Three measures of costs were analysed; costs within the health-care sector, costs due to lost production and total costs (the sum of previous two costs). Costs within the health-care sector arise from diagnostic procedures, operations and rehabilitation and were calculated using the administrative prices paid by a referring hospital to our department (year 2009).

Costs due to lost production were calculated from the number of days of sick leave and the average earnings of the profession [Statistics Sweden; http://www.scb.se] plus the payroll following the human capital approach [14].

All costs are here presented in Euros and we used year 2009 average exchange rate for conversion (EUR 1 = SEK10.6213; [The Swedish Central Bank; http://www.riksbank.se]).

The ethical committee of Lund University approved the study.

### Statistical methods

Descriptive results are presented as median (minimum-maximum) values. The *χ*^2^-test was used to compare groups and the Kruskal-Wallis test were used to analyse costs by place of injury and by attachment to the labour market. The association between the final duration of sick leave and the DASH score at 3, 6 and 12 months, respectively, was analysed by the Kruskal-Wallis test. Pair wise correlation coefficients were estimated between DASH and EQ-5D at study measurements, and between their changes over time. Time to return to work was analysed by Cox-regression analysis stratifying for severity of injury (major injury/severe injury). Factors associated with health-care costs and total costs including costs of lost production were analysed using regression analysis [15].

## Results

### Characteristics of patients and injuries

During the two years of inclusion, 132 patients with a severe and major hand injury were treated at our department. The eighty-seven patients not included in the study and the 45 patients included are summarised in Table 1. The median age for the 45 patients included in the study with a severe and a major hand injury was 42 years (16–64) and the majority of the patients were men (Table 1). The non-dominant hand was injured slightly more often (n = 24, 53%) than the dominant hand (n = 21, 47%). In all patients (n = 132) treated during the study period the monthly distribution showed a slight variation with a peak in May and June. The weekly distribution showed a peak for Friday and Saturday.

**Figure 1:**
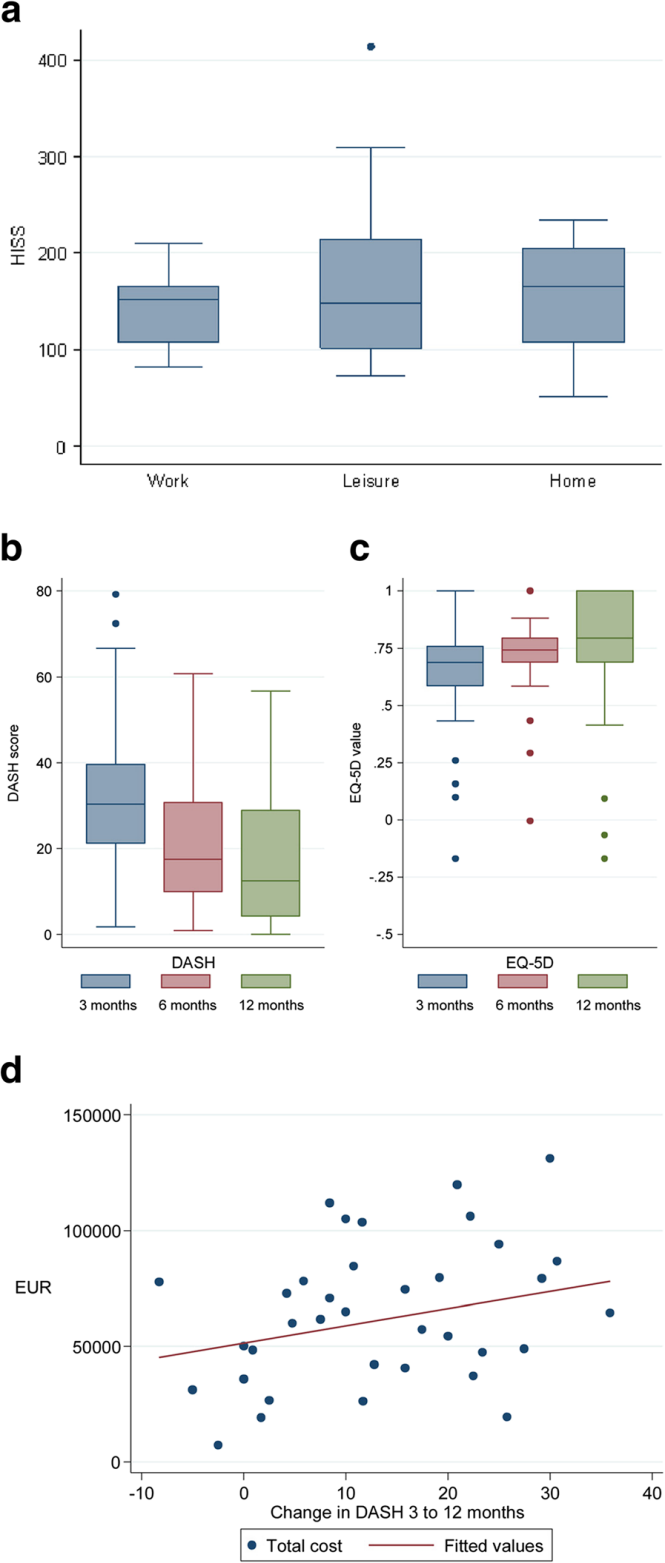
Hand Injury Severity Score (a), DASH scores (b), EQ-5D (c) and costs related to change in DASH score (d) in the 45 patients with a severe and major hand injury.

The injuries of the patients included and followed during the first year after trauma were classified as major in 36 patients and as severe in nine patients based on the HISS (Table 1). Scores of HISS did not differ between the patients injured at work, at home or during leisure activities (Figure 1a) The patients were classified into four groups according to the type of injury: 1. amputation (n = 13, 29%) at any level; 2. complex injury (n = 18, 40%) with a combination of fractures, tendon injuries and vessel injuries 3. major nerve injury/Full house in the forearm (n = 13, 29%); and 4. burn injury, which only occurred in one patient (2%). In almost half of the patients (n = 21, 47%) a reconstruction of the blood vessels was done by microsurgery.

The most common place of injury was outside of work (n = 29; 64%). The injury mechanisms were equally divided in: caused by a machine (n = 12, 27%), by a saw (n = 11, 24%) or by glass (n = 10, 22%). In most of the patients (27; 60%), the injury was a cut (Table 1).

The patients were classified (Statistics Sweden, Socio-economic classification system) regarding to their occupation into four groups: manual workers, non-manual employees, self-employed and non-classified (e.g. students, early retirement, long sick leave). Information about occupation was available in all 45 patients. Among these, four (9%) were not active in the labour market (two students, one early retirement, one long-term sick leave). The majority of the patients had a manual work (28; 62%) and few were self-employed (2; 5%; Table 1). Half of the injuries (15/30; 50%) for manual workers and self-employed were workplace injuries compared to 7% (1/15) for non-manual workers and students. Leisure injuries dominated for the latter group (11/15; 73%; overall difference *χ*^2^ p < 0.01). Patterns associating type of injury with the employment status could not be identified, probably due to lack of statistical power. Seventeen people were still on sick leave at 12 months (Table 1).

### Disability of the arm, shoulder and hand (DASH), and quality of life (EQ-5D)

The median (interquartile range, IQR) DASH score of the 45 patients decreased significantly over time; i.e. at three months 30 (2–80), at six months 17 (1–61) and at 12 months 12 (range, 0–57) (Figure 1b; Kruskal-Wallis p < 0.01). The median DASH scores were higher at each measurement for patients with the longest sick leave and the difference between groups was significant at each measurement (Table 2). The quality of life, measured by the generic EQ-5D instrument, differed at 3, 6 and 12 months (Figure 1c; Kruskal-Wallis p = 0.02). As the DASH-score was decreasing over the study period, the EQ-5D value increased as expected, but the correlation between changes in DASH and EQ-5D 3–12 months and 6–12 months, respectively, was not significant at conventional levels −0.26 (p = 0.15) and −0.30 (p = 0.06). The DASH score and EQ-5D at twelve months were also significantly correlated (−0.72, p < 0.01). There was a strong correlation between patient-assessed EQ-5D at 12 months and physician-assessed EQ-5D using DASH score at 12 months (0.67, p < 0.01). Using regression analysis to control for age and gender did not change the overall results (results available on request). Of the 43 persons with employment or studying at injury who were eligible for sickness benefits, 28 (65%) had returned with at least partial activity. Persons who had returned to activity reported significantly higher median EQ-5D values [0.80 vs. 0.69, p < 0.01].

**Table 2:** **Median DASH (IQR) at 3, 6 and 12 months in patients with different length of sick leave in 43 patients eligible for sick-leave**^**a **^**at the time of injury with severe or major hand injuries**

**DASH (month)**	**0-89 days**	**90-179 days**	**180-364 days**	**365+ days**	**p-value **^**b**^
	**n = 5**	**n = 12**	**n = 11**	**n = 15**	
3	13 (2–23)	27 (5–52)	28 (10–41)	40 (14–79)	<0.01
6	9 (1–13)	13 (2–35)	18 (8–37)	31 (5–61)	0.02
12	6 (0–18)	13 (1–39)	8 (3–31)	30 (1–57)	0.03

^a^ Persons active in the labour market or higher level students. ^b^ Kruskal-Wallis test. *IQR*, interquartile range.

**Figure 2:**
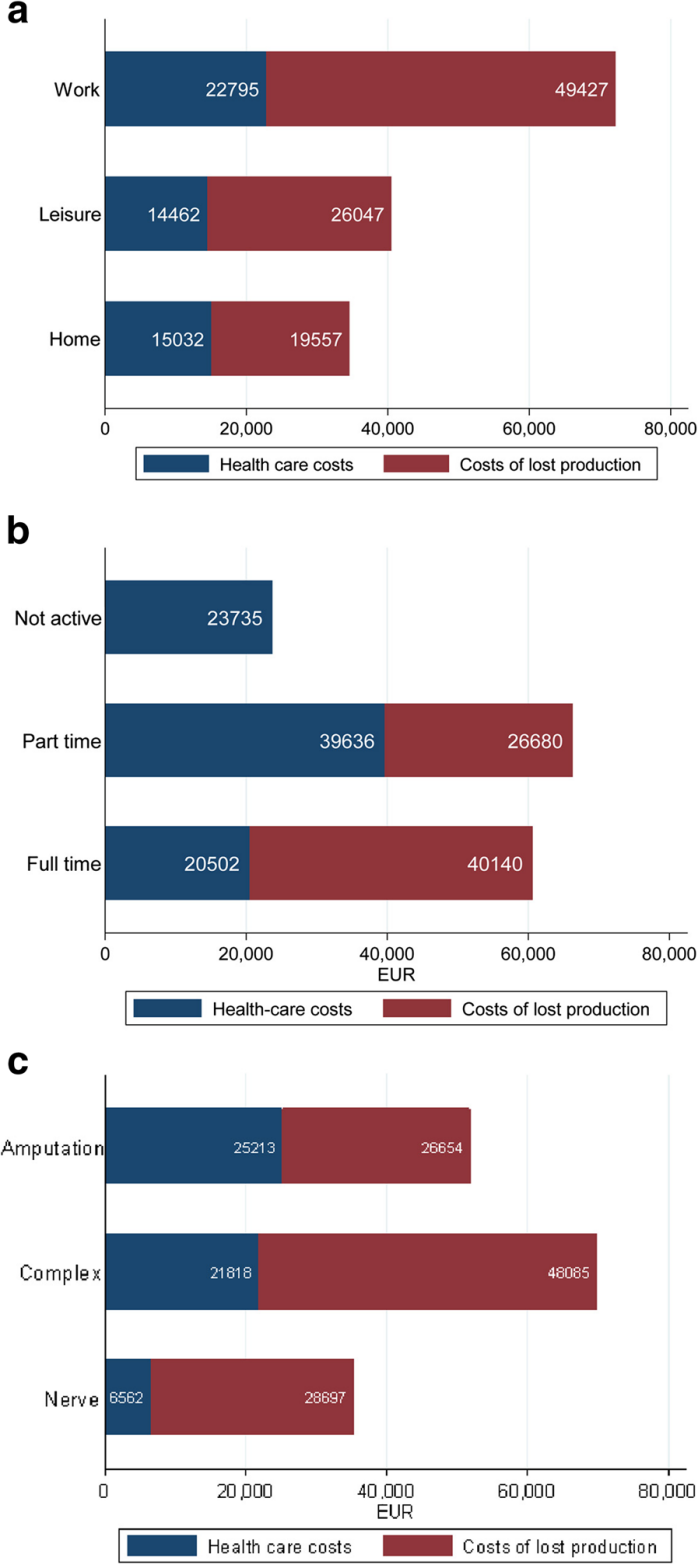
The total costs consist of health care costs (blue) and costs of lost production (red) and divided by place of injury (a) by labour market activity category (b) and by type of injury (c) for the 45 patients with a severe and major hand injury.

Total costs were highest for persons injured at work, although their HISS scores did not differ from the patients that were injured during leisure time or at home (Figures 1a and 2a). The median costs of lost production for patients injured at work was also significantly larger than for patients injured at home or during outdoor leisure activity (Figure 2a; Kruskal-Wallis; health care costs p = 0.46, costs of lost production p = 0.03 and total costs p = 0.02). Costs of lost production had a significant role in total costs of injury, especially for persons injured at work and for persons fully active in the labour market at the time of injury (Figure 2b). Splitting the sample by labour-market status at injury, we found that costs tended to be higher for persons who were partially active mainly because of higher health care costs (Figure 2b). There was a tendency of higher total costs for persons with greater changes in DASH score between 3 and 12 months, although it was not significant at conventional levels (Figure 1d; correlation =0.26; p = 0.11). Patients with amputations and complex injuries had higher health care costs than patients with a nerve injury (Figure 2c). Costs of lost production were also higher among patients with complex injuries. Table 3 shows results by regression analyses that indicated that three factors were significantly associated with higher health care costs: age >50 years (+52%); work injury (+ 40%) and partial labour market activity (+66%). Similarly, total costs were higher for persons injured at work (+34%). Twelve patients had a DASH score > 0 before injury. For each DASH point total costs were 4% lower. Persons who rated high EQ-5D values at 12 months had also lower total costs; e.g. the patients who rated quality of life at 0.8 had 10% lower costs than patients who rated their quality of life at 0.7 (Table 3).

**Table 3:** Factors associated with health care costs and total costs

	**Health care costs**		**Total costs**	
	**Coefficient**	**p-value**	**Coefficient**	**p-value**
Age				
<50 years (reference)				
50+ years	0.52	0.03	NS	
Place of injury				
Home or outdoor leisure (reference)				
At work	0.40	0.07	0.34	0.06
Labour market activity				
Full time working or not in labour market (reference)				
Partially active	0.66	0.04	NS	
DASH value before injury	NS		−0.04	0.07
EQ-5D at 12 months	NS		−1.05	0.01
Constant	9.40	<0.01	11.58	<0.01
Adjusted R-square	0.18		0.24	
N	45		41	

NS variable not significant in equation.

**Figure 3:**
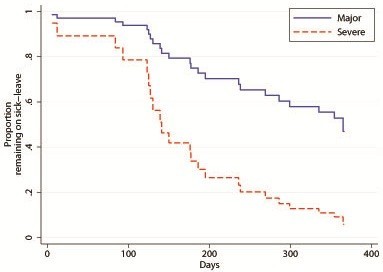
Proportion of patients with a major or severe hand injury remaining on sick-leave at different points in time.

Patients with major injuries had longer duration of sick leave compared to persons with severe injuries (Figure 3). Patients with severe injuries were more likely to return to work [(RR 3.76 (95% CI 1.38-10.22) from Cox regression, controlling for age, gender and presence of nerve injury].

## Discussion

In the present study, with randomly included patients with major or severe hand injuries (HISS score >100; and range 50–100, respectively) and while median HISS scores did not differ by place of injury, work-related injuries had highest total costs. Interestingly, the health care costs of the work-related injuries were 40% higher than those occurring at home or during leisure time controlling for age, DASH at injury and quality of life measured by EQ-5D at 12 months. The total costs were 34% higher in the present patients injured at work. Irrespective of place of injury, costs of lost production constituted the major part of total costs in the patients that worked full time before injury. In contrast, health care costs were +66% in patients working part-time before the injury. We can only speculate as to why in our sample, but it is possible that jobs, where people work part-time, are more likely to be manual and with a possibility of subsequent difficulty to return to work as well as involve greater risks of injury. In addition, the regression analysis also showed that the health care costs were higher for patients with an age > 50 years (+52%). Thus, we had independent cost increases from age and labour market attachment. At one year after injury, 17 patients were still on sick leave and the return to work was related to severity of injury. Patients with severe injuries were near as four times as likely to return to work at a given point in time compared to patients with major injuries (Figure 3). However, several factors may be involved in an injured patient´s ability to return to work and the interconnection of the factors is complex. In a study of costs and utility in the treatment of back and neck pain, the best predictor for return to work when comparing several commonly used health measures was in fact the EQ-5D [16]. Another important factor for return to work is the patients’ sense of coherence in the recovery after hand injuries [17,18] and musculoskeletal pain [19]. We continuously follow our patients with a tentative follow up of at least five years, since the importance of costs and other factors for hand injuries are high-lighted in literature [6,20-23].

The injuries of the present patients could essentially be divided in three equal types; amputations (29%), complex injuries (i.e. combination of fractures, tendon, vessel and nerve injuries; 40%) and major nerve injuries, including full house, in the forearm (29%), while a burn injury was only present in a single patient. The health care costs were higher in the patients with amputations and complex injuries than in the patients with nerve injuries, in spite of those full house injuries were included in the latter category. There was a need for microsurgical reconstruction of blood vessels in almost half of all the cases, which may increase health care costs. Interestingly, all cases with amputations needed microsurgery, while only 3/18 cases (17%) of the complex injuries required microsurgery. In accordance with previous reports [5], the health care costs were only a minor part of the total costs for nerve injuries (i.e. > 80% were lost production). The corresponding percentages of lost production were even lower for the other two main categories (amputations 51%; complex injuries 69%). In the light of these results showing proportionately high costs of lost production, especially for nerve injuries, cost-efficient interventions leading to increasing return to work should have priority [8]. The percentage of lost production (i.e. sick leave) is relevant when considering in on which patients resources for rehabilitation and return to work should be directed. The major part of total costs for nerve injuries were lost production indicating that the return to work should have priority in that category of patients. Nevertheless, the top priority of action is prevention of hand injuries, and this study supports interventions concentrated not only against work-related injuries, but particularly also towards injuries occurring during leisure, e.g. during “doing-your-self activities” [7].

All patients were considered to be fully mobilized at three months and allowed to use their hands without restrictions and thus we report DASH at 3, 6 and 12 months. Patients with longer sick leave had higher DASH scores, but DASH scores improved (i.e. decreased) significantly over time in all patients (Table 2). The improvement of DASH from 3 to 12 months was clinically relevant (median score improvement >10 [24]). In accordance with DASH scores, EQ-5D values also improved (i.e. increased) over time. The findings that patients who rated high EQ-5D values at 12 months had lower total costs are logical, since they had returned to work. There was a tendency of a positive association between increased improvement in DASH score between 3 and 12 months and total costs. One explanation may be that patients with high initial DASH score had a greater potential for improvement, but also were probably more costly in terms of health care resource need and sick leave.

A limitation of this study was that we randomly selected patients with a severe or major hand injury and not all patients during the study period. Our goal was to include as many patients as possible during the study period. These patients came randomly during day/night, weekdays and months and we (i.e. the authors) were also dependent on colleagues for including patients. Sometimes patients were not asked by the hand surgeon on call to participate in the study. However, we could not detect any differences in age, gender, weekday or month of injury between the included and not included patients (Table 1). However, there was a slight overrepresentation of major injuries among the included patients. The strength is the meticulous follow up of patients of costs of injury as well as the other aspects of outcome, such as the influence of sense of coherence [17,25].

We used an episode-perspective to assess the size of costs and to discuss associations between costs, place of injury and patient reported outcomes. It is not a traditional cost-of-illness study, which calculates the burden of illness born by health care and other sectors, but typically it lacks more detailed patient information, including outcomes of treatment. Thus, our approach provides more information of direct clinical interest.

## Conclusions

We conclude that hand injuries at work were most costly both in terms of health care and costs of lost production. This finding is interesting since the severity, i.e. HISS, of work-related injuries did not differ from injuries occurring at home or during leisure and also in view of the fact that work environments have constantly improved over the last decades.

### Consent

Written informed consent was obtained from the patient for publication of this report and any accompanying images.

## Abbreviations

DASH: Disability arm shoulder and hand; EQ-5D: EuroQol 5 dimensions a measure of quality of life; HISS: Hand injury severity score

## Competing interests

The authors declare that they have no competing interests related to the article.

## Authors’ contributions

RC and LD designed the study, RC and HER collected all data, RC, HER, KSC and LD analysed data and all authors participated in the interpretation of data and writing the manuscript. All authors approved the final manuscript.

## Pre-publication history

The pre-publication history for this paper can be accessed here:

http://www.biomedcentral.com/1471-2458/13/501/prepub
